# Separation of Damage Mechanisms in Full Forward Rod Extruded Case-Hardening Steel 16MnCrS5 Using 3D Image Segmentation

**DOI:** 10.3390/ma17123023

**Published:** 2024-06-20

**Authors:** Lars A. Lingnau, Johannes Heermant, Johannes L. Otto, Kai Donnerbauer, Lukas M. Sauer, Lukas Lücker, Marina Macias Barrientos, Frank Walther

**Affiliations:** Chair of Materials Test Engineering (WPT), TU Dortmund University, Baroper Str. 303, D-44227 Dortmund, Germany; johannes.heermant@tu-dortmund.de (J.H.); johannes.otto@tu-dortmund.de (J.L.O.); kai.donnerbauer@tu-dortmund.de (K.D.); lukas.sauer@tu-dortmund.de (L.M.S.); lukas.luecker@tu-dortmund.de (L.L.); marina.macias@tu-dortmund.de (M.M.B.)

**Keywords:** damage mechanism, fatigue, focused ion beam scanning electron microscopy, pores, resistance measurements, axial–torsional fatigue, electron channeling contrast imaging, scanning transmission electron microscopy, atomic force microscopy

## Abstract

In general, formed components are lightweight as well as highly economic and resource efficient. However, forming-induced ductile damage, which particularly affects the formation and growth of pores, has not been considered in the design of components so far. Therefore, an evaluation of forming-induced ductile damage would enable an improved design and take better advantage of the lightweight nature as it affects the static and dynamic mechanical material properties. To quantify the amount, morphology and distribution of the pores, advanced scanning electron microscopy (SEM) methods such as scanning transmission electron microscopy (STEM) and electron channeling contrast imaging (ECCI) were used. Image segmentation using a deep learning algorithm was applied to reproducibly separate the pores from inclusions such as manganese sulfide inclusions. This was achieved via layer-by-layer ablation of the case-hardened steel 16MnCrS5 (DIN 1.7139, AISI/SAE 5115) with a focused ion beam (FIB). The resulting images were reconstructed in a 3D model to gain a mechanism-based understanding beyond the previous 2D investigations.

## 1. Introduction

The operational behavior of components produced by forming technology depends largely on their properties [1]. While machining mainly affects the surface and the boundary layer of the components by up to a few micrometers, forming affects the entire material’s volume. Depending on the magnitude and direction of the stress state during forming, microstructural changes are induced. These in turn affect properties like the yield strength, ultimate tensile strength, hardness, residual stresses and forming-induced ductile damage. Ductile damage refers to the nucleation, growth and coalescence of pores during plastic deformation [2]. This results in a deterioration in the effective mechanical properties. Modifications to the forming process, such as introducing back pressure or changing the shoulder opening angle in cold extrusion, can lead to a reduction in damage and open up the possibility of improving the mechanical properties [3]. Full forward rod extrusion is a forming process used to produce mechanical components such as shafts and bolts. Meya et al. [4] observed that radially superimposed stresses during bending reduce the number of blowholes. Depending on the stress level, these local material discontinuities can occur both during the various forming stages and in service up to significant length scales relevant to material or component failure. Understanding the relationships between the material properties, the microstructure and the operational behavior is therefore of great importance. This importance arises from the ability to select process parameters, considering the criteria of design and functionality, service life, and service load adaptation, which have a positive effect on component performance in terms of energy absorption in notched bar impact tests and life in high-cycle fatigue (HCF) tests. A similar observation has been made during back-pressure extrusion [5]. Higher back pressures reduce the hydrostatic stress state and inhibit shrinkage growth [6], which improves the fatigue life in HCF tests. However, previous research has focused on fatigue behavior after hot forming or axial and torsional fatigue. The low-cycle fatigue (LCF) behavior under axial–torsional loading after full forward extrusion has not been investigated. This combination of forming-induced ductile damage and fatigue is of great economic and environmental importance. A significant number of component failures can be attributed to LCF damage, where the cyclically induced stress exceeds the yield strength, leading to irreversible plastic deformation of steel [7] and non-ferrous alloys [8].

A number of parameters such as the strain rate or strain amplitude have a significant influence on LCF behavior [9,10]. In addition, defects and the near-surface microstructure in particular have a significant influence on LCF fatigue properties. Hardness changes due to work hardening, induced residual stresses or different phase fractions, as well as non-metallic inclusions and the general microstructure, have been investigated. Forming-induced ductile damage occurs in the form of pores in the matrix material or at non-metallic inclusions, leading to local stress increases [11,12]. The resulting material or component failure under service conditions can be attributed to inhomogeneities [13], which have been addressed by various authors in their studies on the correlation between fatigue performance and defects, as the defects are determined by the threshold condition for small crack nuclei at the defect tip [14,15]. In addition, a correlation has been shown between the torsional fatigue limit and the threshold for non-propagating branched cracks. This leads to a multiplication of dislocations and a reduction in the average grain size, which affects the grain boundaries associated with the intervening particles. As a result, dislocation blockage occurs, leading to significant microstructural changes that can have a significant impact on fatigue properties [16]. A fundamental analysis of the effects of room-temperature pre-straining and subsequent LCF loading on steel was performed by Sonsino [17]. Other studies have examined the influence of cyclic loading on material properties after severe plastic deformation processes such as equal channel angle pressing [18]. These included multi-stage fatigue tests of up to 15,000 cycles on hot-rolled steel sheets and fatigue studies of damage-induced effects after hot-rolling with up to 25,000 cycles [19,20]. Teschke et al. performed fatigue tests in the range of 10 to 10^6^ cycles on hot-rolled sheets [21]. Moehring and Walther [22] investigated the influence of forming-induced ductile damage in the LCF range under torsional loading. The influence on forming-induced ductile damage in the LCF range under axial loading was investigated by Langenfeld et al. [12], who showed a significant impact of forming-induced damage on the fatigue performance. In other studies, this influence was also confirmed for axial–torsional loads [23].

Therefore, it is important to extend metallographic characterization methods and combine them with mechanical testing methods as well as structure-sensitive measurement techniques to investigate the influence of forming-induced ductile damage on performance. In addition, non-destructive investigation methods such as computed tomography (CT) are not available for condition characterization due to the microscopic size of the initial forming-induced ductile damage and the density of the steel being examined [24]. For this purpose, focused ion beam scanning electron microscopy (FIB-SEM) investigations with atomic force microscopy (AFM) measurements and a self-developed 3D volume model approach are correlated with the mechanical testing methods to better understand the damage mechanisms. A similar approach has been used by Otto et al. [25] to visualize brittle phases in solder joints to better understand their distribution and the influence on the fatigue and corrosion properties. Other work has focused on carbon-based materials [26] or the influence of voids and inclusions in steels [27], where 3D microstructure imaging has provided new insights into material behavior. Regarding the suitability of the 3D volume reconstruction, the work of Osuch et al. [28] shows that 3D volume reconstruction is suitable for the visualization of carbides in steel materials. Furthermore, the influence of the selected detectors of the SEM on the images and the subsequent segmentation is relevant.

**Figure 1:**
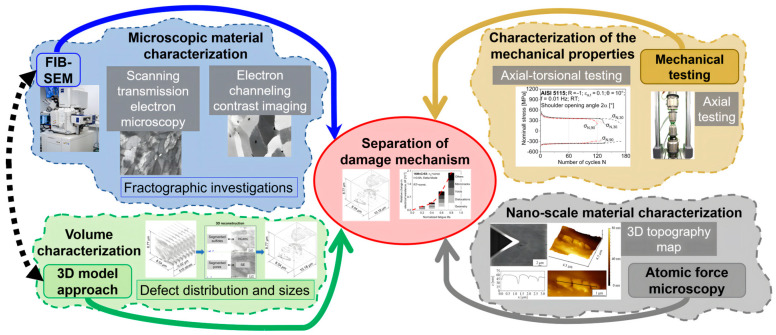
Workflow of the separation of damage mechanisms of components formed via full forward rod extrusion.

Resistometry-based methods will also be used to investigate fatigue damage assessments in terms of the change in electrical resistance due to pore formation or growth [29]. The long-term goal is to quantify the various influencing factors and to separate the damage mechanisms, especially with regard to forming-induced ductile and fatigue-induced damage. The coupling of microstructural analyses and performance characterizations with respect to fatigue properties, as well as the goal of separating damage mechanisms, is shown in Figure 1.

## 2. Materials and Methods

### 2.1. Material

The fatigue specimens were fabricated from cylindrical billets of case-hardening steel 16MnCrS5 (DIN 1.7139, AISI/SAE 5115) via full forward rod extrusion. The base material, supplied by Georgsmarienhuette (Georgsmarienhuette, Germany), consisted of rolled and drawn cylindrical rods with a ferrite–pearlite microstructure. The chemical composition is shown in Table 1.

No additional heat treatment was applied to the material. In the extrusion process, cylindrical billets with an initial diameter (d_0_) of 30 mm and length (l_0_) of 71 mm were forced through a die to reduce their diameter, resulting in an extrusion strain (ε_extr_) of 0.5. This specific extrusion strain induced hydrostatic stress in the forming zone, leading to the development of forming-induced ductile damage. The diameter of the billet after extrusion (d_1_) was observed to be 23.4 mm.

To control the amount of damage developed, different shoulder opening angles were considered. Specifically, shoulder opening angles of 2α = 90° and 2α = 30° were used during the extrusion process. The choice of the shoulder opening angle influences the hydrostatic stress states at the center axes, thereby affecting the level of the forming-induced ductile damage. The full forward rod extrusion process was performed on a triple-acting hydraulic drawing press (HZPUI, SMG, Mannheim, Germany) with a maximum punch force of 2600 kN and a punching speed of 10 mm/s. The billets were coated with Beruforge 179 (Carl Bechem, Hagen, Germany), a MoS_2_-containing coating lubricant, and the extrusion process was conducted at room temperature.

**Figure 2:**
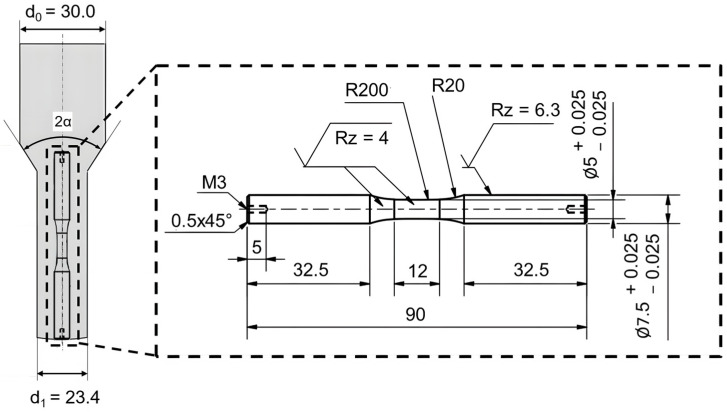
Diagram of fatigue specimen geometry and removal area from the component formed via full forward rod extrusion (all dimensions in mm) based on Möhring [22].

The specimen geometry was designed to maintain comparable levels of strain hardening and residual stress even when subjected to different forming process parameters [3]. The specimens were produced by machining the formed semi-finished products. Hering [3] demonstrated that machining does not significantly affect the damage state or the distribution of forming-induced ductile damage within the specimen. The geometry of the specimens used in this study, along with the extraction position of the specimens from the workpiece after full forward rod extrusion, is detailed in Figure 2.

### 2.2. Fatigue Testing Setup and Methods

**Figure 3:**
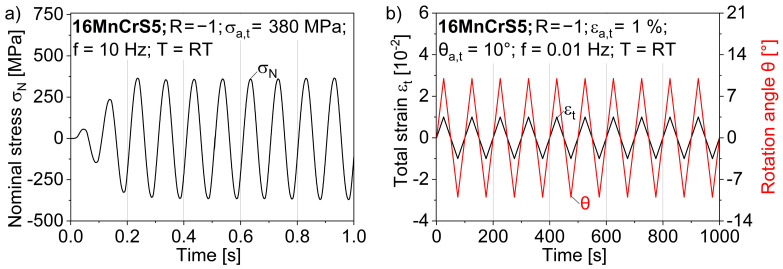
(**a**) Load collective for uniaxial fatigue tests; (**b**) total strain and rotational angle collectives for axial–torsional fatigue tests.

In order to separate the ductile-forming-induced damage from the fatigue-induced damage and to analyze their interaction, constant amplitude fatigue tests were performed on an Instron 8801 servo-hydraulic fatigue-testing system (Instron, Norwood, MA, USA) at a stress amplitude of σ_a_ = 380 MPa with a stress ratio of R = −1 and a test frequency of f_ax_ = 10 Hz (Figure 3a). Axial–torsional fatigue tests were performed using a Walter + Bai, LFV-T250 T2500 HH servo-hydraulic axial–torsional testing system (Walter + Bai, Loehningen, Switzerland). These tests were performed at a test temperature of T = 20 °C. The total strain amplitude was set to ε_a,t_ = 1.0%, with a strain ratio of R = −1 and a test frequency of f_axto_ = 0.01 Hz, along with superimposed torsion controlled by an angular amplitude of θ = 10° with a test frequency of f = 0.01 Hz (Figure 3b). The specimens were clamped using hydraulic chucks, with care taken to ensure that the specimens were clamped with a specimen clamping area of at least 25 mm and in the same position on the chuck to prevent twisting and to ensure comparable test conditions.

**Figure 4:**
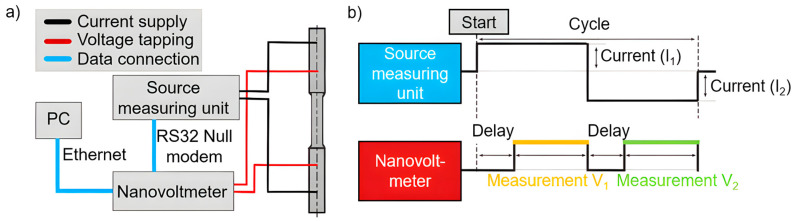
(**a**) Schematic representation of the resistance measurement system and (**b**) the schematic illustration of the ‘delta mode’.

In order to record damage development during fatigue testing, resistivity-based, structure-sensitive methods in the form of direct current potential drop (DCPD) measurements were used. These were conducted with a newly developed experimental setup which allows for sensitive reproducible measurements due to a constant contact pressure and a constant measuring point distance. The electrical current was applied using a Keithley 2602B specific source meter (Keithley, Cleveland, OH, USA) and the electric voltage was measured by using a Keithley 2182A nanovoltmeter (Figure 4a).

The innovative combination of a source meter and voltage measurements allows for the implementation of the ‘delta mode’. In this mode, a highly constant current is applied for 0.09 s, followed by a polarity reversal of the signal, which triggers a nanovoltmeter reading at each polarity (Figure 4b). Therefore, any thermoelectric offsets caused by contact can be avoided. The sampling rate of the resistance-based measuring system is 5.74 Hz. The self-developed software used for data acquisition and system control was specifically programmed for this experimental setup and measurement application. The height of the current is important for electrical resistance measurements. A high current strength leads to an improved measurement accuracy, but simultaneously promotes specimen heating. Through the one-factor-at-a-time method (OFAT), all important adjustable measuring parameters were determined. In addition to the current intensity, the duration of the delay (Figure 4b), which describes the delay of the voltage measurement after the polarity reversal, and the number of power line cycles (NPLC), which describes the sampling rate of the grid cycles of the general power grid, were considered (Table 2). An optimal current of I = 0.8 A, an NPLC of 1 and a delay of 0.02 s were determined, which represents a reliable compromise for the measurements [29].

### 2.3. Microstructural Investigations

The following sections describe the methods used for microstructural analyses. This includes image segmentation, quantitative microstructure analysis, and 3D model generation. In addition, electron channeling contrast imaging (ECCI), scanning transmission electron microscopy (STEM) and AFM methods are briefly explained. For these investigations, a Zeiss Crossbeam XB 550 L FIB-SEM (Zeiss, Oberkochen, Germany) was used.

#### 2.3.1. Image Acquisition

For 3D model generation, thin sections were automatically ablated using an FIB, with an image taken every 20 nm using both Inlens and SE detectors. The milling process was carried out using an FIB acceleration voltage of 30 kV and a sample current of 200 nA. For subsequent fine milling, an acceleration voltage of 5 kV and a sample current of 30 pA were used. By selecting the above parameters, significant heating of the sample can be excluded. A layer width of approx. 9 µm was chosen to obtain a meaningful volume in terms of the pore distribution and sulfide structure distribution while minimizing preparation artifacts due to FIB preparation. Since it is not possible to prepare an image free of artifacts, these were removed with the help of deep learning AI image segmentation.

#### 2.3.2. AI Image Segmentation

**Figure 5:**
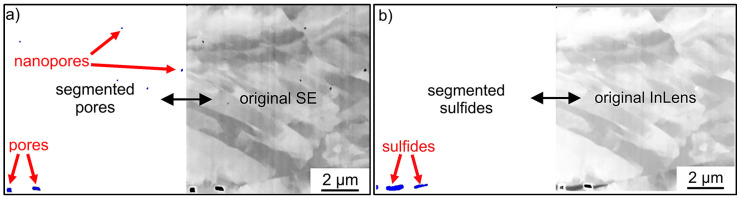
(**a**) Example of segmented pores from an SE image (marked in red) and (**b**) an example of segmented sulfides (marked in red) from an Inlens image of the same slice of the image stack.

Zeiss Zen Analyzer software version 3.5.96.06000 (Zeiss, Oberkochen, Germany) and the Zeiss Zen Intellesis module version 3.5.96.06000 (Zeiss, Oberkochen, Germany) were used for image segmentation. Using a deep learning algorithm, models have been trained to automatically and reliably segment the pores from the SEM SE images. This facilitated the automatic identification and segmentation of the pores (Figure 5a). Using Zen Analyzer’s built-in analysis capabilities, a range of valuable information such as the number, area, coordinates, and geometric properties (e.g., diameter or elliptical semi-axis) could be directly extracted without the need for a specially programmed algorithm.

For sulfide segmentation, a similar approach was used with the Zeiss Zen Analyzer software and the Zen Intellesis module version 3.5.96.06000 with the advantages and features mentioned above. However, SEM Inlens images were used for the segmentation of the sulfide structures instead of the SEM SE images (Figure 5b). To validate the segmentation results, the segmented images were independently compared with the original images by several individuals. Additionally, the image stacks were processed independently by several individuals to compare the quantitative results. The order of the image stacks was also changed to eliminate errors in the algorithm.

#### 2.3.3. Three-Dimensional Model Generation

**Figure 6:**
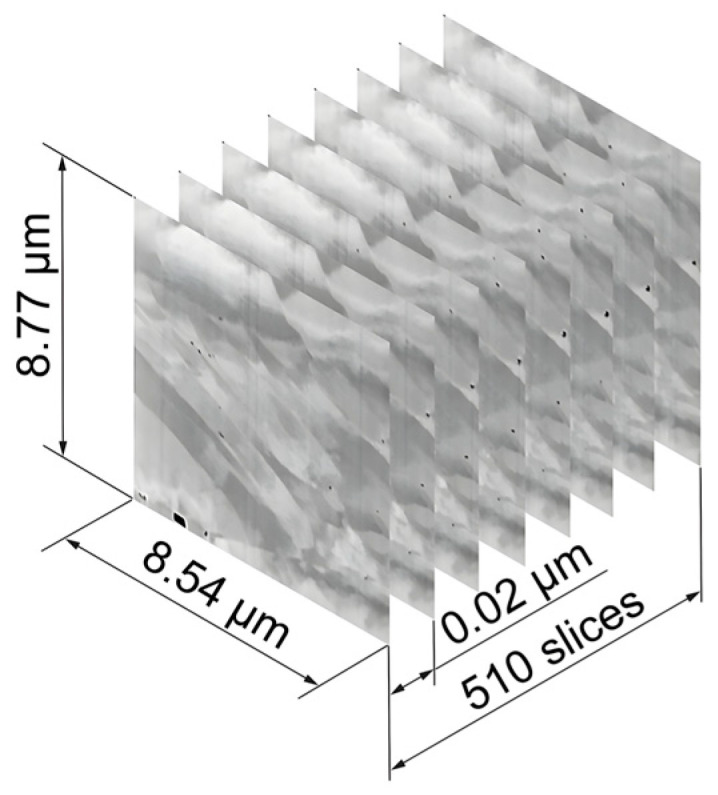
Processing of the FIB-SEM Inlens images.

Figure 6 shows the first steps in processing the FIB-SEM SE images. A region with a low density of artefacts was selected from the 510 images to construct a 3D model measuring 8.54 × 8.77 × 10.18 µm^3^. Automated image cropping was performed using the open-source software FIJI ImageJ version 1.54f. The pores were then segmented from the image stacks using Zen Intellesis version 3.5.96.06000. The pixels were then converted to voxels using FIJI ImageJ and several small mesh models were generated and saved in .obj format. A mesh enabler was used to create a solid model from these mesh structures, and all the parts were assembled into a final model using Autodesk Inventor computer-aided design software version 2024 (Autodesk, San Francisco, CA, USA). The model was then rendered to improve its visual representation. The same workflow was used to create a 3D model of the sulfide distribution. This method is also described in detail in [25] for precipitation reconstruction in brazed joints.

#### 2.3.4. Electron Channeling Contrast Imaging (ECCI) and Scanning Transmission Electron Microscopy (STEM)

**Figure 7:**
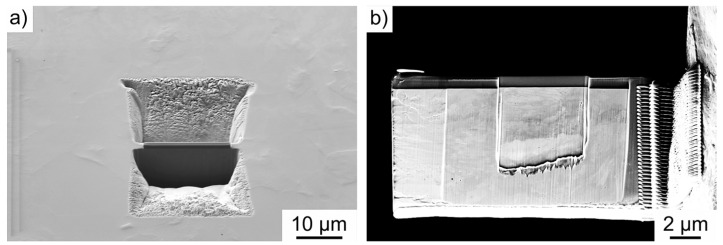
(**a**) Illustration of the milling process and (**b**) further lamella preparation for the STEM investigations.

For the ECCI investigations, the specimens were prepared according to the specific requirements. An acceleration voltage of U = 25 kV and a specimen current of I = 1 nA were used. Dislocations can be visualized by a specific orientation of the electron channels. Other defects such as pores can also be clearly seen from these images and can be correlated with the other methods. For STEM studies, the specimens were prepared according to the specific requirements for STEM analysis (Figure 7a) by preparing the specimens to a thickness of less than 100 nm for transillumination (Figure 7b) using the FIB.

The milling process was performed with an FIB acceleration voltage of 30 kV and a sample current of 200 nA. For the subsequent fine milling of the FIB, an acceleration voltage of 5 kV and a sample current of 30 pA were used. For the microscopic examination of the FIB lamella, an acceleration voltage of U = 30 kV and a sample current of I = 1 nA were used. These parameters were found to be optimal for imaging pores and dislocations with the highest possible resolution and contrast. In order to correlate the results of the STEM studies with the 3D SE studies, the images were then segmented and analyzed in ZEN-Intellesis with respect to the pores.

#### 2.3.5. Atomic Force Microscopy (AFM) Measurements

For an in-depth assessment of the shape, topography and separation of pores and cracks inside sulfides in the nanometer range, AFM investigations were performed for selected specimens, using a piezo-based AFM (LiteScope, Nenovision Brno, Czech Republic). AFM investigations were performed in tapping mode with Akiyama probes as single-pass measurements. The topography was studied with setpoints of around 5 Hz at a resonance frequency of the probe of around 45 kHz. AFM investigations were performed under vacuum inside a Crossbeam XB 550 L FIB-SEM (Zeiss, Oberkochen, Germany). This ensures stable oscillation at resonance and also helps to find the region of interest. After or even during acquisition of AFM data, SEM images can be acquired using this setup. Post-processing of AFM data was achieved using the free open-source software Gwyddion Version 2.63.

## 3. Results and Discussion

### 3.1. Axial–Torsional and Uni-Axial Fatigue Behavior

**Figure 8:**
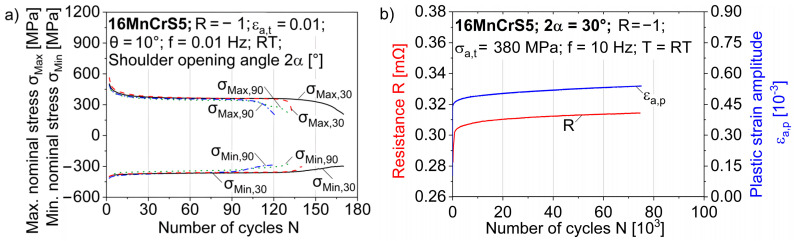
(**a**) Shoulder-opening-angle-dependent material responses of axial–torsional tested specimens and (**b**) an exemplary representation of the absolute resistance and the plastic strain amplitude of an axial tested specimen with a shoulder opening angle of 2α = 30°.

The influence of forming-induced ductile damage, which is dependent on the process parameter of the shoulder opening angle 2α, on the fatigue behavior in LCF has already been demonstrated for torsional and axial stresses [12,29,30]. The influence of forming-induced damage at superimposed axial–torsional stresses has not yet been determined. The material response for the strain-controlled fatigue tests with a total strain amplitude of ε_a,t_ = 1.0%, a stress ratio of R = −1 and an angular amplitude of θ = 10° is qualitatively comparable for the two shoulder opening angles of 2α = 30° and 2α = 90°. The material response can be seen in the form of the maximum and minimum nominal stresses in the axial direction. There is also a decrease in the yield stress in the compressive region, which can be attributed to known phenomena such as the Bauschinger effect. Cyclic softening can be observed for both damage states, particularly in the first few cycles before a linear decrease in stress occurs (Figure 8a). Cyclic softening, which occurs primarily in the first 15 cycles, was also observed under axial loading and is consistent with LCF studies of axial fatigue behavior [12]. This can mainly be explained by the hardening during the forming process in full forward rod extrusion. During cold forming, dislocations accumulate at barriers, such as precipitates and grain boundaries, and form dislocation clusters and interlocks, leading to an increase in dislocation density [16]. Under cyclic loading, the dislocation density decreases due to mechanisms such as dislocation annihilation. The influence of forming-induced ductile damage as a function of the shoulder opening angle can be seen from the number of cycles to failure, N_f_. This is 1.24 times higher for a shoulder opening angle of 2α = 30° than for a shoulder opening angle of 2α = 90°.

With regard to the axial stress states in the HCF regime, initial investigations were carried out by Luecker et al. [29] in combination with the resistance-based methods to quantify the forming-induced ductile damage by full forward rod extrusion. Using the GOM Aramis SRX high-speed DIC system (Zeiss, Braunschweig, Germany), the changes in plastic strain amplitude could be analyzed and correlated with the electrical resistance measured using the Keithley 2182A nanovoltmeter measurement system (Keithley, Cleveland, OH, USA). The total strain was extracted with the system manufacturer’s software GOM Correlate Professional version 2020 (Zeiss, Braunschweig, Germany) from the DIC measurements using 5 mm virtual gauge line elements in the central section of the specimen (Figure 8b). The change in plastic strain amplitude correlates well with the absolute value of the resistivity, so that the damage accumulation can be recorded using structure-sensitive measurement methods to describe damage mechanisms such as pore coalescence or crack propagation. It should be noted that the test shown was stopped at approx. 75% of the expected number of cycles to failure N_f_.

### 3.2. Microstructural Investigations

#### 3.2.1. Fracture Analysis

**Figure 9:**
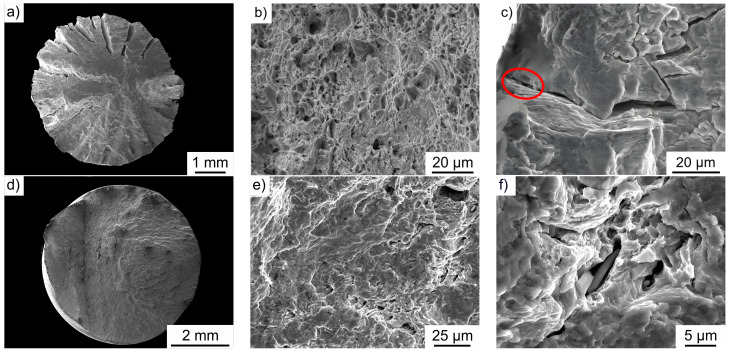
(**a**) Macroscopic fracture surface of a full forward rod extruded specimen with a shoulder opening angle of 2α = 30° after axial–torsional fatigue stress and fractographic images of (**b**) ductile fracture areas and (**c**) the crack initiation near MnS inclusions (marked in red); (**d**) macroscopic fracture surface of a full forward rod extruded specimen with a shoulder opening angle of 2α = 30° after axial fatigue stress and fractographic images of (**e**) ductile fracture areas and (**f**) MnS inclusions on the fracture area.

To analyze the damage mechanisms and the interaction between fatigue loading and forming-induced ductile damage, the fracture surfaces of the axial–torsional- and axial-loaded specimens were examined microstructurally. The fracture surface of the axial–torsional-loaded specimen in the LCF regime is shown in Figure 9a. The test was performed under strain-controlled conditions with a strain ratio of R = −1, a total strain amplitude of ε_a,t_ = 1.0%, and a test frequency of f = 0.01 Hz. In addition, an angular amplitude of θ = 10° was superimposed at a test frequency of f = 0.01 Hz. The number of cycles to failure was N_f_ = 133. The fracture surface shows several crack initiation points typical of LCF regimes and consists of a mixture of characteristic fatigue and ductile fracture areas (Figure 9b). The cyclic damage progressed to final fracture of the specimen at an angle of approximately 45° to the maximum normal stress in the volume. The degradation of the material was supported by the development of cracks in the bulk in the longitudinal direction, comparable to Möhring [23,30]. It was found that the crack initiation was preferentially located at MnS inclusions or at pores close to MnS inclusions, and thus, the forming-induced ductile damage played a central role in crack initiation as well as crack propagation (Figure 9c,f). The cyclic damage development under axial–torsional loading was based on different mechanisms, as evidenced by different fracture morphologies, especially radial inward cracking. The influence of MnS inclusions and pores on crack propagation was demonstrated for both damage states independent of the degree of initial forming-induced ductile damage. Furthermore, it was found that crack initiation also occurs preferentially at MnS inclusions close to the surface and pores close to MnS inclusions, and thus the initial forming-induced damage is of highest relevance in terms of crack initiation, as has already been shown in investigations for torsional stresses [30].

Figure 9d shows an example of the fracture surface of an axially loaded specimen in the HCF range. The stress amplitude is σ_a_ = 380 MPa at a stress ratio of R = −1 and a test frequency of f = 10 Hz. Both ductile and characteristic fatigue fracture areas are seen (Figure 9e). Similar to the axial–torsional-loaded specimens, the crack initiating influence of MnS inclusions and pores in the vicinity of MnS inclusions can also be seen under axial loading (Figure 9f). Near-surface MnS inclusions and pores in the vicinity of MnS inclusions are particularly critical for crack initiation, which is consistent with the results obtained for axial stresses in the LCF range from other studies [12].

#### 3.2.2. Atomic Force Microscopy (AFM) Pore Investigations

**Figure 10:**
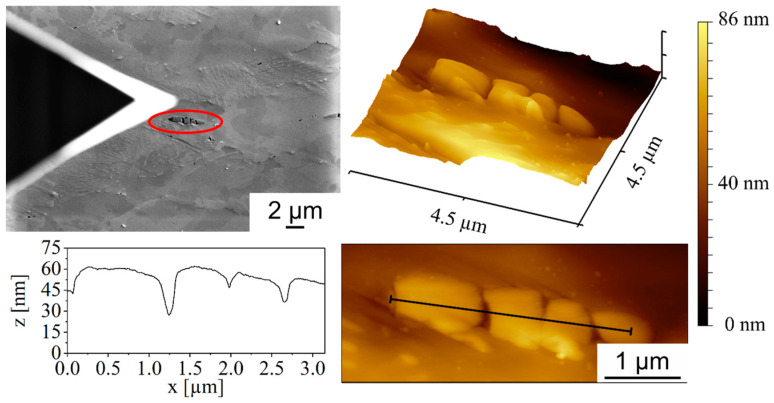
AFM measurement of pores inside MnS inclusions (marked in red) of full forward rod extruded case-hardening steel 16MnCrS5 (DIN 1.7139; AISI/SAE 5115) before fatigue testing and the corresponding AFM line profile recorded with Akiyama probes in single-pass tapping mode.

The measurement of sulfides via AFM allows for a high-resolution characterization of the surface morphology on the nanoscale and thus a clear characterization of the topographic features and pore formation. The measurements, performed in single-pass tapping mode with Akiyama probes, could be merged with SEM images to correlate the topography maps with SE images (Figure 10).

**Figure 11:**
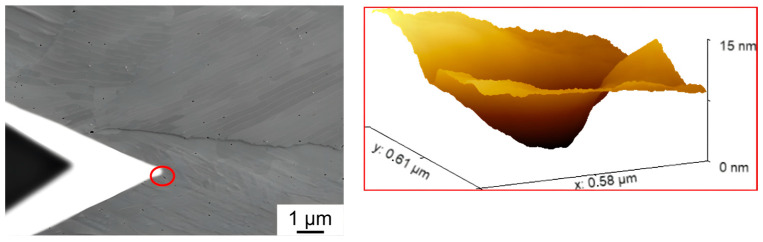
AFM measurement of nanopores (marked in red) in the perlite phases of full forward rod extruded case-hardening steel 16MnCrS5 (DIN 1.7139; AISI/SAE 5115) before fatigue testing.

In particular, the influence of full forward rod extrusion on the sulfide structures in terms of pore formation could be studied. In the SE image, the fracture of the sulfide structures, already suspected from Hering’s investigations [3], could be observed. Based on the line profile shown in Figure 10, the clear fracture of the sulfides with the associated pore formation could be validated using AFM. In addition to the formation of pores in or near the sulfide structures, the formation of nanometer-sized pores was observed for the first time (Figure 11). These are preferentially located within the perlite phases. They are mainly located between the cementite clusters and are only a few nanometers in size. These nanopores can also explain the discrepancy between the 2D pore measurements and the density measurements according to Hering [3], as these nanoscale pores were not detected in the pore measurements due to their small size.

#### 3.2.3. Advanced Scanning Electron Microscopy (SEM) Investigations

**Figure 12:**
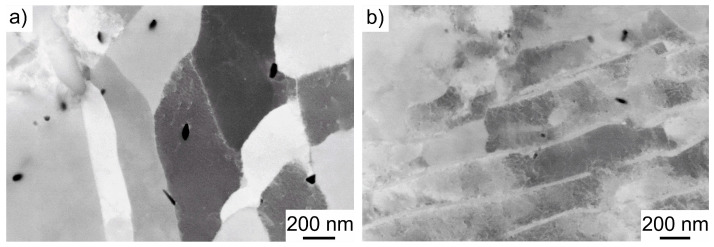
(**a**) ECCI images of nanopores in the ferrite and (**b**) perlite phases of full forward rod extruded case-hardening steel 16MnCrS5 (DIN 1.7139; AISI/SAE 5115) before fatigue testing.

Using advanced SEM methods such as ECCI, it is possible to visualize the nanopores described above at a high resolution and to obtain information about the crystal structure and defects within the material. This allowed the AFM measurements to be validated and the dislocation structures to be visualized. A quantitative determination of the dislocation densities is not possible due to the high degree of deformation caused by full forward extrusion. However, a qualitative comparison is possible, which shows that the dislocation density is increased in both ferrite (Figure 12a) and pearlite (Figure 12b). Furthermore, the pore distribution in both ferrite and pearlite was confirmed to be comparable to the AFM measurements.

**Figure 13:**
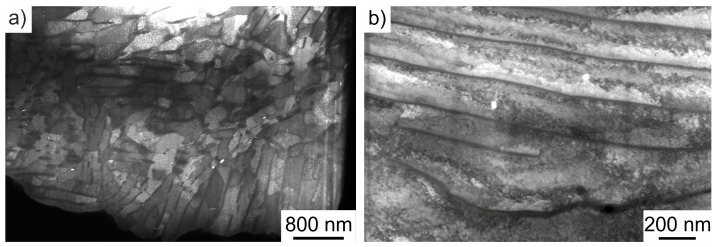
(**a**) Overview STEM image of the FIB lamella and (**b**) STEM image of the dislocations in the pearlite phase of full forward rod extruded case-hardening steel 16MnCrS5 (DIN 1.7139; AISI/SAE 5115) before fatigue testing.

Additional STEM images provide more information about the dislocation density and pore distribution. The overview image in Figure 13a shows the typical ferritic–pearlitic microstructure including very bright areas which can be identified as pores. These are distributed in both the ferritic and pearlitic phases in a similar manner to the microstructural investigations via ECCI. Nanopores inside the pearlite between the cementite lamellae can be seen in Figure 13b. The increase in the strain hardening density as a result of the forming process visible in the ECCI images is confirmed in the STEM images.

#### 3.2.4. Three-Dimensional Analysis

**Figure 14:**
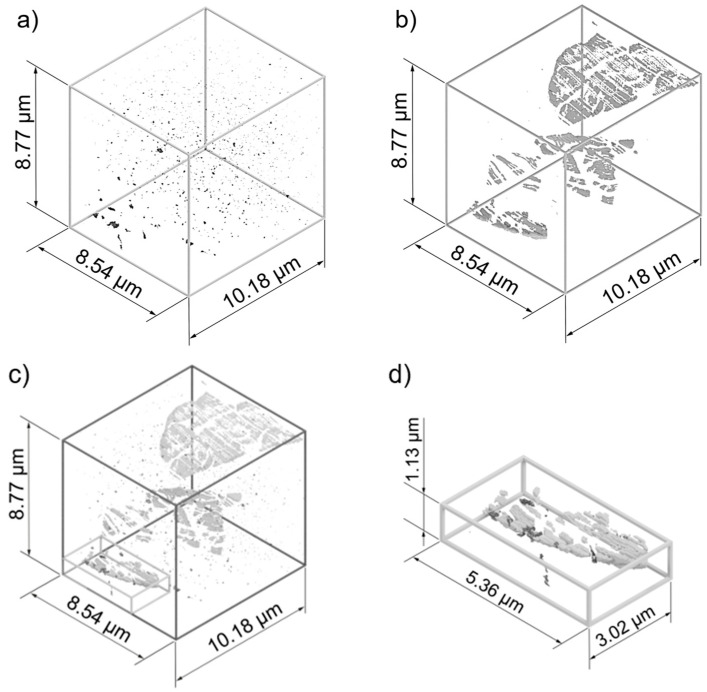
Rendered 3D CAD of (**a**) pore distribution, (**b**) sulfide distribution, (**c**) combined distribution of pores and sulfides, and (**d**) magnified section of the combined distribution of pores and sulfides of full forward rod extruded case-hardening steel 16MnCrS5 (DIN 1.7139; AISI/SAE 5115) before fatigue testing.

In order to study the influence of the pores and sulfide structures on the fatigue performance and to understand the resulting damage mechanisms, multiple rendered 3D CAD models are shown in Figure 14. Figure 14a shows the rendered 3D CAD model segmented and generated from 510 SE images. It can be seen that the pores are distributed throughout the representative volume and are not only found in the sulfide structures as previously thought [3]. In addition, the 3D CAD model also shows nanopores present in both the ferrite and pearlite phases, which was validated via ECCI, STEM and AFM measurements. The nanopores can explain the discrepancies between the density measurements and the 2D analysis methods from previous studies [3]. Compared to the 2D analysis methods used so far, information about the pore distribution can be used to draw new conclusions about crack propagation and crack initiation, especially for complex axial–torsional loading cases. The volume fraction of the pores is about 0.13%.

Fractographic investigations and previous studies [12,22,23,30] have shown that MnS inclusions also play a significant role in pore formation during the full forward rod extrusion process and in fatigue with respect to crack initiation and crack propagation. For this reason, a 3D CAD MnS model was generated from the segmented 510 Inlens images (Figure 14b). For the first time, it is possible to study the shape of sulfides in full forward extruded specimens. This provides valuable new insights into the interpretation of previous 2D results. The results show that the sulfide structures form plate-like structures as a result of the full forward rod extrusion process and generate strong microstructural notching effects in terms of crack initiation and crack propagation. The deformation limited by the brittle phases cannot be compensated for by the deformation of the matrix and therefore leads to fracture of the sulfides within the matrix both during the full forward rod extrusion process and as a result of fatigue. This results in the formation of large pores in and around the MnS inclusions, which can lead to accelerated crack propagation. The volume fraction of MnS inclusions corresponds to about 0.27%.

Since the 2D investigations carried out so far have mainly examined pores within fractured MnS inclusions, nanopores visualized by various methods have not been considered in other studies. Figure 14c shows a 3D CAD model that includes both pores and MnS inclusions. The assumption made in earlier work [3,30] using 2D methods that pores form within the MnS inclusions could be confirmed with the 3D CAD model. These are the largest pores by volume and are located within or around the MnS inclusions (Figure 14d). However, it can also be seen that the nanopores in particular are distributed throughout the representative volume, and their influence on crack propagation has been neglected or not considered in previous work [12,29,30]. To better understand this quantitatively, the regions or phases in which the pores are located were examined. The BSE images of the MnS inclusions were compared with the SE images of the pores. By making intersections, it was found that approximately 1.9% of the pores are formed within manganese sulfides. However, it should be noted that the pores around the manganese sulfide structures could not be examined using this intersection approach. Thus, the probability of pore formation in the MnS inclusions is significantly higher than in the remaining representative volume or in the ferrite and pearlite phase, considering that the manganese sulfides account for 0.27% of the volume. Thus, the segmentation of MnS inclusions and pores provides new insights into their distribution in the volume as well as separates the damage influences.

## 4. Conclusions and Outlook

The present study has shown that forming-induced ductile damage has a significant influence on the fatigue properties of full forward rod extruded case-hardened steel 16MnCrS5 (DIN 1.7139, AISI/SAE 5115). This has been demonstrated for both stress-controlled uni-axial and axial–torsional strain-controlled fatigue tests. The damage mechanisms were validated by a microstructural analysis using AFM, ECCI and STEM. In addition, advanced metallographic techniques were used to visualize pores and sulfide distributions in 3D for the first time to infer the shape and distribution of defects in the form of pores and MnS inclusions.

With respect to axial–torsional stress, cyclic softening and tensile–compressive asymmetry were observed, which can be attributed to the Bauschinger effect. This was confirmed by ECCI and STEM images of the dislocation structures in the fully forward extruded material, with an increased dislocation density occurring in both ferrite and pearlitic phases. Under cyclic loading, the dislocation density decreases due to mechanisms such as dislocation annihilation, which is reflected in the cyclic softening of the material. It has also been shown that the cyclic axial–torsional fatigue behavior can be improved by adjusting the forming parameters during full forward rod extrusion and the resulting forming-induced ductile damage. The specimens with a shoulder opening angle of 2α = 30° required an approximately 24% higher number of load cycles to failure, N_f_, compared to a shoulder opening angle of 2α = 90°.

For uni-axial stresses in the HCF range, a resistometry-based method was used to separate forming-induced ductile and fatigue damage. The local deformation behavior was analyzed using DIC. By using the newly developed experimental setup, the measurement scatter of the resistometry-based measurement was minimized by more than a factor of 10^2^ compared previous measurements using conventional DCPD systems. The electrical resistance was correlated with the deformation behavior, which was measured using a proprietary test setup. The cyclic softening that occurs under axial–torsional stress could also be observed from the plastic strain amplitude in the axial-tested specimen. Thus, the resistometry-based method provides a good opportunity to quantify forming-induced ductile damage and to record damage accumulation in situ during the fatigue test to detect damage mechanisms such as pore coalescence or crack propagation. The information provided by the non-destructive measurement technique was used to validate the quantitative determination of the void content, and this technique offers a fast and cost-effective method for quantifying the degree of forming-induced ductile damage in the future.

The influence of pores and MnS inclusions on crack initiation and propagation was emphasized by fractographic studies, as these play a significant role, especially in areas close to the surface. High-resolution AFM measurements of MnS inclusions confirmed the hypothesis that full forward rod extrusion leads to fracturing of the MnS inclusions and thus to pore formation. In addition, AFM was used for the first time to visualize the formation of nanopores in fully forward rod extruded 16MnCrS5 (DIN 1.7139; AISI/SAE 5115). These were found preferentially in the pearlite phases between the cementite clusters, and also in the ferrite. This was confirmed via ECCI and STEM.

By combining FIB-SEM, deep learning AI image segmentation and CAD, high-resolution 3D models of the MnS inclusions and pore distribution were generated for the first time in the full forward rod extruded case-hardening steel 16MnCrS5 (DIN 1.7139; AISI/SAE 5115). It was found that the pores are distributed throughout the representative volume and not, as previously thought, only in the sulfide structures. This is mainly due to previously undetected nanopores in both the ferrite and pearlite phases, as confirmed via ECCI, STEM and AFM measurements. By visualizing the distribution of the MnS inclusions, this influence could also be considered separately from the pores with respect to crack initiation and propagation, as a significant influence of these was determined in the fractographic investigations. The platelet-shaped sulfide structures also show a microstructural notch effect, as the brittle phases cannot compensate for the deformation of the matrix. By combining the two 3D models, it was possible to determine that the largest pores were located in and around the MnS inclusions. Considering the pore distribution in the present phases, it can be seen that about 1.9% of the pores occur in MnS inclusions, and thus the probability of pore formation within a MnS inclusion is about ten times higher than in the remaining representative volume or in the ferrite and pearlite phases, since the sulfide structures account for only 0.27% of the representative volume. Mechanisms such as decohesion between MnS inclusions and the surrounding ferrite–pearlite matrix could not be considered in this work because only the intersections between pores and sulfides were compared.

The segmentation of MnS inclusions and pores thus provides new insights into their distribution in the volume as well as the separation of damage mechanisms. This will allow future conclusions on crack propagation and crack initiation, especially for complex axial–torsional load cases. With this information, future forming processes must be designed to minimize forming-induced ductile damage in order to improve the tensile as well as fatigue properties, as it has a significant impact on them. This concerns both the suppression of pore formation and pore coalescence. The combination of in situ damage measurements during fatigue tests with the developed resistometry-based measuring system and the determination of pores, MnS inclusions and crack development in 3D can be used in the future to carry out damage-controlled forming processes.

Further investigations of short-term crack growth and defect development or damage accumulation due to different fatigue states are the next logical steps to develop a deeper understanding of the influence of initial forming-induced ductile damage using the 3D models. In the future, in situ tests inside the SEM will be performed to evaluate the microstructural damage development. In addition, the studies will be extended to the very high cycle fatigue range using a Rumul Gigaforte 50 to cover all application-relevant cycle ranges.

## Figures and Tables

**Table 1:** Chemical composition of the case-hardening steel 16MnCrS5 (DIN 1.7139, AISI/SAE 5115) in wt.%.

		C	Si	Mn	P	S	Cr	Fe
**16MnCrS5**		0.14	<0.40	1.10	0.010	0.027	0.80	bal.
DIN EN 10084	Min.	0.14	-	1.00	-	0.020	0.80	bal.
	Max.	0.19	0.40	1.30	0.035	0.035	1.10	bal.

**Table 2:** One-factor-at-a-time method (OFAT) plan for the determination of the ideal parameters of the resistometry-based measuring system.

Parameter	Default Setting	Variation	Selected Parameter
Current [Ampere]	1.0	0.2; 0.4; 0.6; 0.8; 1.2; 1.4; 1.6; 1.8; 2.0	0.8
NPLC	1	1; 2; 3; 4	1
Delay [s]	0.01	0.01; 0.02; 0.05; 0.10; 1.00	0.02

## Data Availability

All raw/processed data required to reproduce these findings are available from the authors upon reasonable requests.
